# Expanding Predictive Capacities in Toxicology: Insights from Hackathon-Enhanced Data and Model Aggregation

**DOI:** 10.3390/molecules29081826

**Published:** 2024-04-17

**Authors:** Dmitrii O. Shkil, Alina A. Muhamedzhanova, Philipp I. Petrov, Ekaterina V. Skorb, Timur A. Aliev, Ilya S. Steshin, Alexander V. Tumanov, Alexander S. Kislinskiy, Maxim V. Fedorov

**Affiliations:** 1 Syntelly LLC, Moscow 121205, Russia; muhamedzhanova@syntelly.com (A.A.M.); steshin@syntelly.com (I.S.S.); tumanov@syntelly.com (A.V.T.); kislinskiy@syntelly.com (A.S.K.); 2 Moscow Institute of Physics and Technology, Moscow 141700, Russia; 3 Medtech.Moscow, Moscow 119571, Russia; philip.i.petrov@gmail.com; 4 Infochemistry Scientific Center, ITMO University, Saint-Petersburg 191002, Russia; skorb@itmo.ru (E.V.S.); aliev@infochemistry.ru (T.A.A.); 5 Kharkevich Institute for Information Transmission Problems of Russian Academy of Sciences, Moscow 127994, Russia

**Keywords:** hackathon, cheminformatics, neural networks, deep learning, toxicity, machine learning, gradient boosting

## Abstract

In the realm of predictive toxicology for small molecules, the applicability domain of QSAR models is often limited by the coverage of the chemical space in the training set. Consequently, classical models fail to provide reliable predictions for wide classes of molecules. However, the emergence of innovative data collection methods such as intensive hackathons have promise to quickly expand the available chemical space for model construction. Combined with algorithmic refinement methods, these tools can address the challenges of toxicity prediction, enhancing both the robustness and applicability of the corresponding models. This study aimed to investigate the roles of gradient boosting and strategic data aggregation in enhancing the predictivity ability of models for the toxicity of small organic molecules. We focused on evaluating the impact of incorporating fragment features and expanding the chemical space, facilitated by a comprehensive dataset procured in an open hackathon. We used gradient boosting techniques, accounting for critical features such as the structural fragments or functional groups often associated with manifestations of toxicity.

## 1. Introduction

Drug discovery, a sophisticated and arduous process, is driven by the promise of finding novel therapeutic agents that can effectively combat disease without compromising patients’ safety [1,2,3,4,5]. However, the journey from the initial identification of the compound to the successful launch of a safe drug is riddled with challenges [6,7,8]. A pivotal aspect of this journey is predicting and understanding a drug’s toxicity profile. Efficient prediction of toxicity endpoints can substantially streamline the drug development process, ensuring that potentially harmful agents are identified and eliminated early in the research [7,9,10,11,12,13,14,15,16]. Ensuring the availability of top-notch reference data is crucial for advancing, authenticating, and applying both in vitro and in silico methods that aim to minimize and substitute the use of animals in evaluations of toxicity [17,18,19,20].

However, the task is made more formidable by two pressing issues. First, there is a stark lack of comprehensive and reliable toxicity data for many chemical compounds. Given the multitude of chemicals used in industries, only a fraction (less than 0.1%) has been thoroughly tested for their toxic effects [21,22,23,24]. This scarcity of data limits our understanding and poses potential unseen risks. Second, the available toxicity data often exhibit significant variance, which can be attributed to factors such as differences in the experimental conditions, biological models used, and inter-species variability [25,26,27]. This inconsistency further complicates the prediction process and stresses the need for standardized testing procedures.

The landscape of drug discovery has undergone significant evolution over the years, with the advent of computational techniques playing a pivotal role. Cheminformatics has been at the forefront of this transformation [28,29,30]. With the increasing complexity of the challenges faced, cheminformatics hackathons have surged in prominence, serving as crucibles for innovative algorithmic and data-driven approaches. Notably, these events have often transcended the traditional definitions of hackathons, with many of them not even explicitly termed as such. A comprehensive overview of the activities undertaken in this domain over the past decade is provided in Table 1.

Broadly, these events can be classified into two categories. The primary category encompasses activities where the participants engage in project-driven endeavors without the expectation of a substantial financial recompense. The overarching objective here is to foster a symbiotic exchange of knowledge between the participants and organizers. Those involved predominantly accrue benefits in terms of establishing professional connections, acquiring novel skill sets, and gaining hands-on experience in groundbreaking projects. The outcomes from such endeavors often manifest as scholarly publications, enhancements to existing software tools, or the inception of novel ideas with the potential to evolve into standalone projects. This confluence of conferences, workshops, and open-source project development has led to the emergence of such unique platforms. Noteworthy events falling within this category include the RDKit UGM [31], D3R Grand Challenge [32], MATDAT18 [33], Drugathon [34], and CATMOS [35].

The secondary category includes contests that offer financial incentives to the top performers. These events are delineated by well-defined and mutually agreed-upon evaluation metrics, ensuring clarity in the adjudication process. Predominantly, such competitions are geared towards the deployment of machine learning techniques, given the amenability of these methodologies to rigorous and formalized evaluations. A standard paradigm in these events is assessing the proficiency of the participants’ models based on a specified metric or set of metrics, which quantitatively gauge the predictive or analytical power of the model in question. One of the most renowned platforms hosting such competitions is Kaggle [36], which has garnered worldwide recognition for facilitating a diverse array of data science contests. Within the realm of cheminformatics, competitions such as Nomad2018 Predicting Transparent Conductor [37], Novozymes Enzyme Stability Prediction [38], Predicting Molecular Properties [39], and Bristol–Myers Squibb—Molecular Translation [40] underscore the increasing intersection of machine learning and chemistry.

In this article, we aimed to detail the methodology used in organizing the hackathon and to elucidate the outcomes that were subsequently achieved. In April 2023, the Syntelly team hosted a meticulously designed three-day hackathon open for participation by dedicated teams, which addressed the prediction of toxicity endpoints [41]. The participants were tasked with utilizing open-source data for curating a dataset and building a machine learning model that could accurately predict the toxicity endpoints of small molecule compounds. Syntelly’s hackathon was more than a mere competition; it was a strategic endeavor to confront the persistent issue of data scarcity in the domain. By incorporating both the tasks of data curation and model building into the hackathon, Syntelly aimed to highlight the symbiotic relationship between comprehensive data collection and the refinement of machine learning models in the field.

**Table 1:** A compilation of events within the past decade that align with the definition of a hackathon in the realm of cheminformatics.

Event	Topic	Year
RDKit UGM [31]	An annual symposium centered around the RDKit cheminformatics library, wherein attendees engaged with presentations, facilitated dialogues, exchanged insights, and familiarized themselves with recent advancements in cheminformatics algorithms, pipelines, and databases. The concluding day was structured as a hackathon, focusing on resolving practical challenges pertinent to RDKit and KNIME.	2012–2023
D3R Grand Challenge [32]	This endeavor was designed to hone computational methodologies for estimating ligand–protein interaction energies and predicting their binding conformations. Notably, the challenge garnered support from multiple leading pharmaceutical entities, contributing data pertaining to the docking structures.	2015–2018
MATDAT18 [33]	During the hackathon, contributions spanned the development of machine learning models for material classification and crafting structure–performance correlations, complemented by advancements in computational strategies in the domain of force fields and descriptors.	2018
Nomad2018 Predicting Transparent Conductor [37]	This initiative sought to devise a strategy for the systematic engineering of highly efficient conductors based on metal sesquioxides. Participants were tasked with predicting both the band gap and atomic formation energy for conductors consisting of the combination of aluminum, gallium, and indium.	2018
Predicting Molecular Properties [39]	Participants were tasked with leveraging nuclear magnetic resonance data to craft an algorithm proficient in forecasting the spin–spin interaction constants between paired atoms.	2019
Bristol-Myers Squibb—Molecular Translation [40]	The focal point of this competition was the optical recognition of chemical structures, subsequently transcribed to InChI format. The synthetic datasets provided encompassed distorted images of chemical compounds.	2021
CATMOS [35]	An international coalition was commissioned to predict five distinct endpoints: EPA and GHS categorizations, dichotomous toxicological outcomes, and pinpoint estimations of LD50 for acute oral toxicity in rodents.	2021
Drugathon [34]	In this engagement, attendees were encouraged to showcase their prowess in molecular modeling and drug discovery paradigms. Submissions entailed proposals for putatively active chemical entities. Following a rigorous selection process by the orchestrating entity, BioSolveIT, the most promising submissions were synthesized to validate their biological activity. Upon successful validation, BioSolveIT extended co-authorship opportunities for a publication in a reputable, peer-reviewed journal.	2022–2023
Novozymes Enzyme Stability Prediction [38]	The challenge mandated the creation of a machine learning model adept at predicting an enzyme’s thermostability, inclusive of its single-amino acid variants. The metric of thermal stability was equated to the enzyme’s melting point.	2022–2023

A notable aspect of the hackathon was the criteria set used for evaluation. In formulating the assessment benchmarks, Syntelly adopted a hybrid approach. While the evaluation primarily focused on quantitative metrics to gauge the efficacy and accuracy of the predictive models, it also encompassed non-quantitative assessment categories. These included factors such as the relevance of the dataset, the coherence and clarity in the presentation of results, and the overall interpretability of the proposed models. The evaluation strategy adopted by Syntelly was multifaceted, placing emphasis on both technical expertise and the coherent conveyance of the results and methodologies. Through this approach, there was a notable improvement in the model’s metrics for established endpoints. Additionally, the initiative led to the compilation of a database encompassing diverse toxicity data, showcasing the potential of short-term dedicated collaborative efforts in addressing challenges within cheminformatics.

## 2. Results

It was expected that the participants’ solutions would not be completely comprehensive. In the rigorous environment of academic studies, these solutions often lack an intricate comparison of diverse algorithms involving metrics cited in the existing literature and subsequent empirical data, a practice common in exhaustive scientific writings. However, the outcomes derived are akin to those outlined in brief reports or flash presentations from conferences.

The first place was taken by the MML team (Nick Kutuzov and Sergey Novikov from the Moscow Institute of Physics and Technology), who proposed a solution based on Catboost [42]. Using a combination of Daylight fingerprints, MACCS keys, and several standard molecular descriptors, it was possible to improve models predicting the lethal dose in mice and rats at different doses (Table 2). Daylight fingerprints were generated in 8, 128, and 256 bits with path lengths of 3 and 7, after which, the bits were combined. Among the standard descriptors, the number of atoms of the first 50 elements, the TPSA, the molar mass, and the number of heteroatoms and valence electrons were used. The comparison was carried out with the metrics of the multitask model of a fully connected neural network, the architecture of which was considered in an earlier publication by Sosnin et al. [43], and the metrics with five-fold cross-validation were presented on Syntelly’s website [44]. The MML team claimed that they were not able to achieve high-quality using language models or graph featurizers in combination with gradient boosting or neural networks, which Catboost produces with fingerprints and molecular descriptors.

The Billy QSAR team (Ruslan Lukin and Boris Pyakilla from Innopolis University) took second place and presented results based on the Tox21 dataset. The team used Catboost based only on RDKit descriptors and MACCS keys. It has been shown that using such a simple model can be superior to language models [45] for the classification problem using the Tox21 dataset (Table 3). The Billy QSAR team also tried using graph neural networks, in particular ALIGNN [46]; however, CatBoost showed the best results.

The evaluation of the performance of the fingerprint-based CatBoost and fragment-based XGBoost [47] algorithms was conducted by using RMSE metrics for regression tasks and ROC AUC for classification tasks. An examination of these metrics revealed a notable enhancement in performance compared with models previously published in academic literature and those available on the TOXRIC website [48]. An observable improvement was also documented in the metrics of existing models hosted on our Syntelly website (Table 4). An in-depth analysis of six datasets, each exceeding 10,000 samples, indicated a trend of improvement in the metrics or a marginal decline, with the exception being the mouse subcutaneous LD50 target. Notably, the voluminous nature of the datasets used for model development surpassed the sizes of the datasets customarily used, underscoring the extensive exploration of the chemical space. In the context of datasets ranging from 1000 to 10,000 samples, the XGBoost Fragments model outperformed renowned models in the majority of instances. For datasets comprising up to 1000 samples, victories were observed for both the CatBoost FP model and the XGBoost Fragments model in several instances. It is noteworthy that XGBoost Fragments emerged as the superior model in 55% (16/29) of the evaluated cases.

In the domain of classification tasks, an analysis of the XGBoost Fragments model revealed a propensity for enhancement in the metrics in 64% of instances (Table 5). A comparative evaluation with the CardioTox model, characterized by its intricate architecture, delineated a marginal differential in the performance metrics, highlighting a nuanced landscape of algorithmic efficiency. The applicative capacity of XGBoost Fragments extended to the Tox21 dataset, where an analysis indicated improvements in the metrics in 8 of the 12 cases when juxtaposed with the standard CatBoost FPs and the TranGRU language model. An examination of specific targets, such as eye irritation and eye corrosion, hepatotoxicity, the Ames test, carcinogenicity, and blood–brain barrier penetration, delineated an observable trend of enhancement in the metrics associated with the application of gradient boosting on fragments.

## 3. Discussion

**Figure 1:**
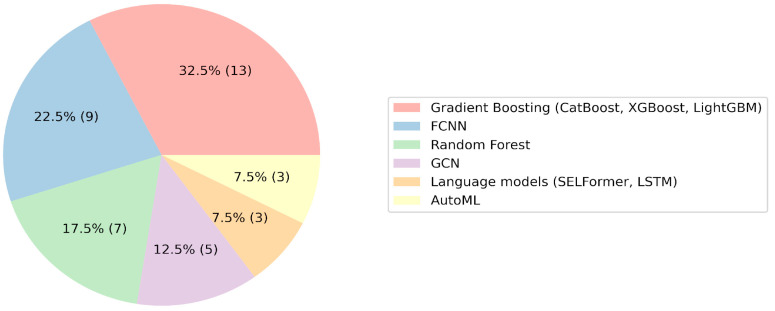
Pie chart of the number of teams that used any machine learning algorithm. The percentages on the slices show the overall preference of the teams for choosing an algorithm; the absolute number of teams is indicated in parentheses.

An examination of the machine learning algorithms used by participants revealed a prevalence of gradient boosting approaches, namely CatBoost, XGBoost, and LightGBM [52]. Fully connected neural networks, graph convolutional networks, and AutoML were also frequently utilized (Figure 1). Conversely, the application of more intricate models, such as LSTM or SELFormer, yielded inconclusive results, as indicated by the participants’ presentations. The hypothesis emerged that gradient boosting could potentially serve as an optimal choice for baseline QSAR models, with standard deep learning approaches not consistently offering marked enhancements. This proposition was corroborated by the performance of CatBoost users, who secured the first and second places, outperforming teams utilizing graph convolutional networks and linguistic models. Other researchers also confirmed the high efficiency of gradient boosting in comparison with other algorithms when applied to problems in chemistry [53].

**Figure 2:**
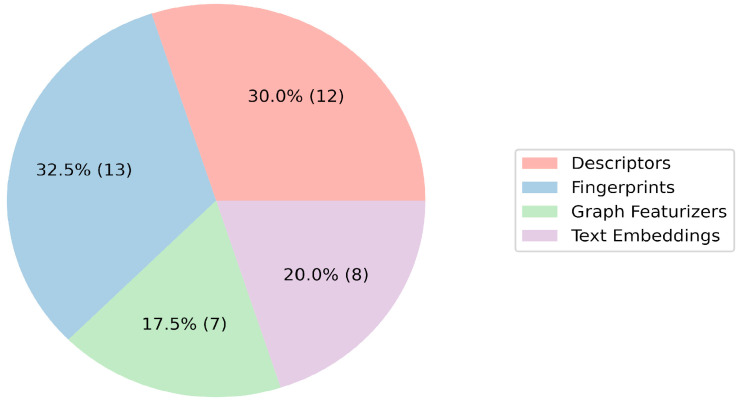
Pie chart of the number of teams that used any group of features. The percentages on the slices show the overall preference of the teams for choosing an algorithm; the absolute number of teams is indicated in parentheses.

**Figure 3:**
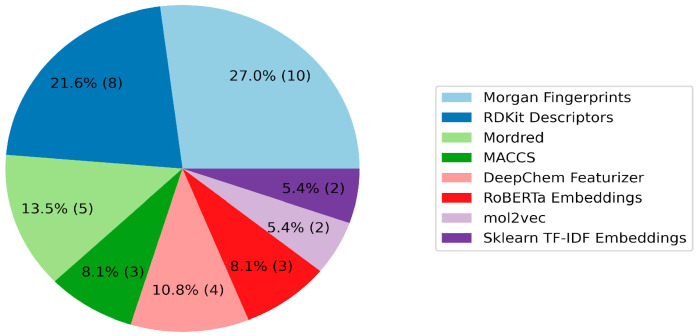
Pie chart of the number of teams that used any features. The features that were used by only one team have been removed from the diagram. The percentages on the slices show the overall preference of the teams for choosing an algorithm; the absolute number of teams is indicated in parentheses.

The role of features in vectorizing molecules is critical in the context of QSAR model construction. The evaluation of the participants’ feature selection processes unveiled a varied utilization of four primary categories: descriptors, fingerprints, graph featurizers, and text embeddings (Figure 2 and Figure 3; Table S1 in the Supporting Information). Classic features (descriptors/fingerprints) saw frequent application; however, a substantial number of teams also used graph and text features. Morgan fingerprints and descriptors from the RDKit library emerged as the predominant individual features. Additionally, the Mordred 1.2.0 package, offering a diverse array of features including topological, electrotopological, and 3D descriptors, was used by five teams. The integration of graph featurizers and text embeddings with gradient boosting algorithms was noted, though significant results remained elusive.

**Figure 4:**
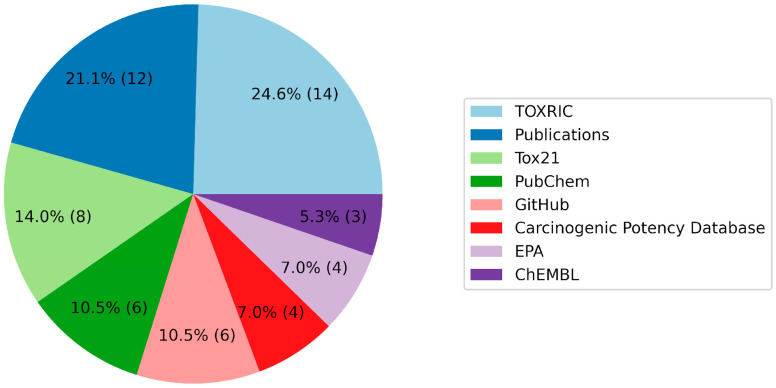
Chart showing the number of teams that used specific sources. Those sources that were used by fewer than three teams were removed from the diagram. The percentages on the slices show the overall preference of the teams for choosing an algorithm; the absolute number of teams is indicated in parentheses.

A review of the databases used by the hackathon’s participants highlighted a pronounced use of TOXRIC, Tox21, PubChem, and academic publications, supplemented by resources available on GitHub (Table 6, Figure 4). TOXRIC’s popularity can be attributed to its user-friendly data download and manipulation capabilities, notwithstanding its limitation of housing multiple datasets with restricted sample sizes. The frequent utilization of academic publications underscores their primacy as sources of experimental data, underscoring the hackathon’s effectiveness in addressing the challenges of data collection. Conversely, the limited use of ChEMBL could potentially be ascribed to the complexities associated with delineating the correlations between on-target biological activities and toxicity parameters.

In the context of the hackathon centered on predictive toxicology, the participants faced rigorous criteria, including extensive data collection, model training, and the effective communication of results. This intensive process saw 27 out of 80 teams successfully completing the challenge. Each contribution unveiled a comprehensive array of insights into predictive toxicology. Included were diverse sets of data and resources, strategies for data gathering, and applications of a variety of machine learning techniques. The results offered detailed analyses of the efficiency and limitations associated with diverse algorithms applied to distinct targets, and illuminated the overarching strategies used by the participants to navigate the complexities of the assigned tasks. A close examination of the 27 submissions revealed a noticeable diversity in the approaches. This highlighted the ability of this hybrid hackathon format to elicit a broad spectrum of solutions, a feature less observed in metric-focused platforms where evaluations, mainly anchored on metrics, often lead to markedly similar solutions. The resulting analysis underscored the diversity of insights and methodologies in predictive toxicology that have surfaced from this event. These findings, subject to further exploration and validation, hold the potential to offer pivotal insights that would be instrumental in propelling advancements in this field.

**Figure 5:**
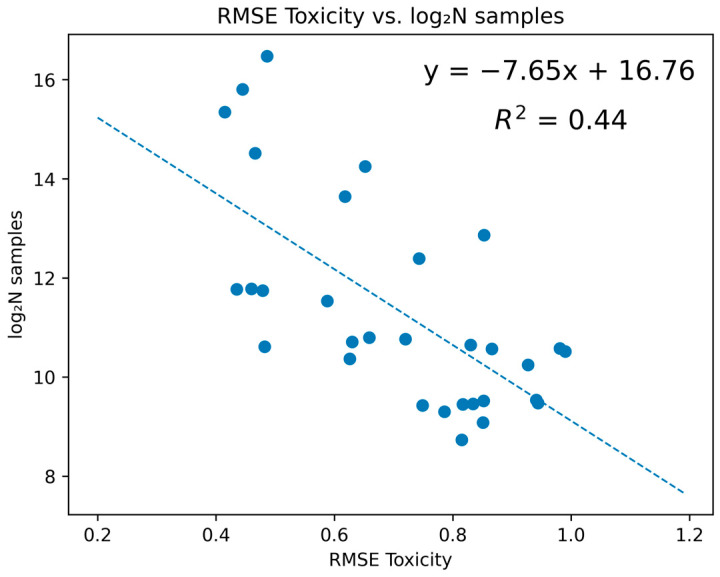
Dependence of RMSE values according to the XGBoost Fragments model on the number of samples in the dataset on a logarithmic scale.

As we moved towards the concluding phase of our investigation, attention was directed towards elucidating the statistical correlation between the model’s quality and the volume of samples incorporated. Preliminary insights underscored the pivotal role of the volume of the sample in fostering the development of robust models (Figure 5).

While acknowledging the integral role of the data’s quality and adept preprocessing, emphasis was also placed on the potential role of expansive datasets in enhancing predictive accuracy. Our observations underscored the potential trajectory towards enhanced predictive accuracy, seen at the threshold of 30,000 samples. This volume was associated with a reduction in error margins, indicated by an RMSE approximating ~0.2, a development that would constitute a significant milestone in the ongoing journey of advancements in predictive toxicology. Our findings aimed to catalyze concerted efforts towards the accumulation of expansive and qualitatively rich datasets, underscoring the symbiotic relationship between the volume of data and the models’ accuracy in the context of predictive toxicology.

## 4. Materials and Methods

### 4.1. Organization of the Hackathon

The center for innovative technologies, Medtech.Moscow, supported the hackathon with a prize fund amounting to approximately EUR 10,000. The event drew participation from 80 teams spanning 47 distinct Russian regions. As expected, a significant portion of participants came from the major cities of the Russian Federation: Moscow (40%) and St. Petersburg (37%). The median age of the participants was 22.5 years, suggesting a predominant representation from undergraduate and graduate student demographics. Collectively, the participants were affiliated with 105 different universities. Teams were restricted to a maximum of five members, with single-member teams being precluded, primarily due to the multifaceted nature of the challenges. Constraints were also set to manage the demands on the expert committee and to ensure adequate computational resources for all teams.

Throughout the hackathon’s duration, daily question and answer sessions were facilitated to address any ambiguities in the problem statement. Moreover, there were two designated timeframes for direct discussions with experts from the fields of cheminformatics and data science. By the hackathon’s end, 27 teams had successfully uploaded the solutions. The logistical aspects, inclusive of the question and answer sessions, expert interactions, and evaluations of the solutions, were managed by Syntelly and Infochemistry Scientific Center from ITMO University.

To support the computational needs, servers provided by Selectel Ltd. (St. Petersburg, Russia) were utilized, operating on a ‘one team—one server’ model to pre-empt potential technical challenges associated with singular computing clusters. The servers’ specifications included an Intel Xeon Processor E5-2630 v4 2.20 GHz (Intel, Santa Clara, CA, USA), an Nvidia Tesla T4 16 GB (Nvidia, Santa Clara, CA, USA), and 64 GB of RAM. Of the participating teams, only 25 (31%) opted to use the servers provided. 

Participants were required to submit their solutions as a zip archive containing:

1. A presentation detailing the solution.
2. A Python-based Jupyter notebook containing:
  - A module taking a test .csv file of molecular SMILES as the input and producing the corresponding predicted endpoints as the output.
  - A complete model training workflow including parameter selection, hyperparameter tuning, and definition of the architecture.
3. Training datasets with the molecular SMILES and the corresponding experimental values, clearly delineating data sources.
4. Any supplementary files necessary for the model’s execution or evaluation purposes.

It was incumbent upon the participants to ensure that the Jupyter notebooks were executable for any valid canonical SMILES generated by RDKit. Non-compliant solutions were subject to penalties, including potential disqualification.

The evaluation metrics for the hackathon were custom-developed. Instead of adhering to a singular metric-based evaluation, akin to platforms such as Kaggle, a broader evaluation framework was implemented. The intention was to foster a spectrum of scientifically rigorous solutions. Consequently, the event resembled more of a scientific competition within a given domain rather than merely an algorithmic contest, providing the participants with a structured framework for creating quantitative structure–activity relationship (QSAR) models.

### 4.2. Solution Evaluation Criteria

1. Dataset quality. Evaluations under this metric did not award additional points. Rather, it eliminated suboptimal submissions. Solutions were penalized for inconsistencies such as the use of synthetic data, attempts at fraud, discrepancies between the source and the data utilized, inconsistencies in the dimensions of the data, the presence of duplicate molecular entities, or a lack of source provision. This metric sought to emphasize the importance of initial data processing; a foundational step commonly practiced by researchers.
2. Model evaluation. Participants were prompted to utilize evaluation metrics either from referenced publications or from the Syntelly models, available on the Syntelly website in the statistics section. For quantitative assessment, any improvement had to surpass a 5% mean value for regression (RMSE) or 3% for classification (ROC AUC) during a five-fold cross-validation. Depending on improvements in the metrics, the models’ multipliers were adjusted. For the metrics available in Syntelly, a comparison with the average values of Syntelly’s benchmarks was required. For non-Syntelly metrics, external benchmark citations were mandated. Notably, minor model improvements deemed to be statistically insignificant were not considered valuable, leading to the establishment of a minimal improvement threshold. If the model fulfilled the condition for improving the quality of the metric, then the model’s multiplier equaled (1 + M/100) for regression and equaled (1 + M/50) for classification, where M is the percentage of improvement in the metric.
3. The assessment process for the models incorporated two primary factors: the extent of the dataset and the uniqueness of the samples. For the dataset’s extent, a point-based system was used, attributing 1, 0.75, and 0.5 points for the first, second, and third positions, respectively. In instances of tied positions, the points were distributed evenly among the tied participants. Zero points were assigned for datasets that were not within the top three positions.

In assessing a sample’s uniqueness, the focus lay on the inclusion of unique molecular structures absent in other participants’ submissions. Similar point allocations to the extent of the dataset were used. This criterion ensured streamlined manual verification of the solutions and incentivized participants to broaden the data pool, addressing the common issue of data insufficiency in QSAR models.

4. Difficulty in obtaining data. The challenge associated with data acquisition was acknowledged and quantitatively assessed. Points were allocated based on the complexity of the data collection process. One point was assigned for scraping data without an API from at least one source, downloading via API from three or more sources, or manual searches of five or more sources. Downloading via API from at least one source or a manual search of at least three sources scored 0.5 points. A manual search of one source scored 0.25 points. Collecting good data is an important task as part of building a machine learning model, so we aimed to reward participants if the participants spent a large amount of time collecting good data.
5. Number of sources of experimental data. In consideration of the significant variability inherent in experimental toxicity data, models incorporating data from multiple sources were favored. A source was defined as a database or an aggregation of publications with over 1000 chemical structures. Points were allocated as per the number of sources, with a model incorporating over five sources receiving 1 point, four or five sources receiving 0.75 points, two or three sources receiving 0.5 points, and one source receiving 0 points.
6. Number of predicted toxicity models. Models were evaluated on the basis of the range of toxicity endpoints predicted. Each endpoint was scored individually, with the total points computed cumulatively, incorporating the respective multipliers.
7. Quality of presentation of the solution. It was not enough to simply send files with the models and the metrics’ results; an essential quality for a researcher is the accurate presentation of their results. This was particularly important, considering the educational value of the project, as many of the participants were students and graduate students. The ability to clearly and concisely present the results determines how valuable the participants’ contribution will be to the scientific community. At this point, participants could receive additional points for the thoughtfulness of the solution and a competent methodology for selecting parameters and hyperparameters of the model. The Jupyter notebook should have had: (1) a selection of hyperparameters (more than 10 options were considered) or the number of layers of the neural network (more than three options were considered); (2) a selection of neural network architectures or a selection of machine learning models, additional comments describing each of the blocks; (3) the ease of perception of the laptop and convenient launch. If the laptop could not be started from start to finish, 0 points were given for the work. Each item on the Jupyter notebook was worth 1 point (the maximum number of points for a notebook was 3 points). The presentation had to include: (1) a detailed description of the data collection process, selection of and/or the search for models and descriptors, and the results obtained; (2) the provision of complete information about all models, benchmarks used and the sources, (3) the clarity, adequacy, and consistency of the information presented, free of factual errors. For the presence of factual errors, 0 points were given for the presentation. Each presentation point was worth 1 point (the maximum number of points for a presentation was 3 points).
8. Diversity of the dataset. Models benefitting from a broad chemical space were awarded a point, contingent on the demonstration of the dataset’s diversity, spanning multiple chemical classes.
9. Uniqueness of the space of toxicity indicators. An additional dimension of the evaluation lay in the variation of predicted rates amongst the participants. A point was awarded for the inclusion of unique toxicity endpoints that were absent in other submissions and directly pertinent to molecular toxicity.
10. Interpretability of the model and descriptors used. The capacity for the models to elucidate the toxic effects of molecules, offering insights into the underlying mechanisms, was rewarded with an additional point, accentuating the importance of the model’s interpretability in the context of scientific discovery.

The formula for calculating points was

$$Score=\sum_{n=1}^{a_{6}}A_{1n}A_{2n}A_{3n}\left(a_{3n}+a_{4n}+a_{5n}+a_{8n}+a_{10n}\right)+a_{7}+a_{7}^{\prime }+a_{9}$$

where *Score* is the overall score of the team, *A*_1*n*_ is the quality coefficient of the dataset used to build the model, *A*_2*n*_ is the quality coefficient of the model, *A*_3*n*_ is the coefficient accounting for the length of the dataset, *a*_3*n*_ is the score for the uniqueness of the sample, *a*_4*n*_ is the score for the difficulty of obtaining data, *a*_5*n*_ is the score for the number of sources, *a*_6_ is the number of predicted indicators, *a*_7_ is the score for the Jupyter notebook, $a_{7}^{\prime }$ is the score for the presentation, *a*_8*n*_ is the score for the diversity of the dataset, *a*_9_ is the score for the uniqueness of the space of the toxicity indicators, and *a*_10*n*_ is the score for the interpretability of the model and descriptors used. The score was calculated under the condition that all the required files were downloaded and there was at least one value of *A*_1*n*_ ≠ 0. If at least one of the conditions was violated, then the work of the entire team was assigned 0 points.

### 4.3. Model Preparation

Following the systematic examination of the participants’ solutions, with a spotlight on those of the victors, we elected to prioritize the use of molecular fingerprints and CatBoost with hyperparameter optimization (see S1 in the Supporting Information) via Optuna, and molecular fragments and XGBoost optimized through Grid Search CV as the principal machine learning algorithms. This selection was part of a strategic initiative to develop a refined toxicity prediction model for integration into Syntelly. We used descriptors based on molecular fragments as features with their own implementation, since they are most similar to fingerprints but can be more customized for the toxicity task. It is known that toxicity is often caused by the presence of some functional groups or fragments which subsequently have a negative biological effect due to the metabolism of the fragment or the interaction of the functional group with biomolecules (for example, proteins) [96,97,98]. We collected all the datasets that were presented at the hackathon and aggregated them. The primary aggregation of data for binary classification was carried out as follows: (1) if the activities of molecules from different sources did not match, such samples were excluded from the general database; (2) if the activities of a molecule from different sources coincided, the duplicate record was deleted. The initial aggregation of data for a regression problem was slightly different due to the fact that records for chemical compounds may be duplicated in different databases, and when converting units (for example, between mg/kg and mmol/kg), differences in the final values may occur. (1) All values for a structure for a specific target for all sources were averaged. (2) Values for structure were first averaged within data from one source, then averaged across sources. Finding duplicate SMILES was first carried out by converting them into canonical form using the RDKit module, after which, all compounds that did not contain a carbon atom or contain any element that was not in the list of elements (N, I, As, O, B, Br, F, P, Se, S, C, Cl, and Si) were removed, as they were considered outliers. Next, we filtered out rare fragments based on a (0,1) matrix of the occurrence of a fragment in a molecule; those fragments in which the frequency of fragment’s occurrence was less than 1% were removed. Next, with the remaining fragments, a matrix of the quantitative occurrence of the fragment in the molecule was constructed. Fragments with a total number of occurrences below 2.5% were discarded. We also eliminated fragments that had a high Pearson correlation (r > 0.85) based on their location in the structure. The final features in the form of the number of fragments present in the molecule were used to build a machine learning model. We also selected additional features from the following list from the RDKit library if the frequency of non-zero feature values was greater than or equal to 2.5%: the occurrence of elements in the molecule, the number of non-hydrogen atoms, the number of bonds of each type (single, double, triple, and aromatic), the number of valence electrons, the number of rotational bonds, TPSA, logP, LabuteASA, Kappa1, Kappa2, Kappa3, SlogPVSA, and SMRVSA EStateVSA (Table S2).

## 5. Conclusions

This study underscored the utilization of gradient boosting and data aggregation as pivotal components in enhancing the efficiency of predictive models. The integration of an expansive dataset, enriched by the contributions garnered during the hackathon, facilitates a nuanced understanding of the intricate dynamics underpinning toxicological predictions. Structural fragments or functional groups, often implicated in manifestations of toxicity, were emphasized as critical features that augment the predictive precision of models.

The hackathon served as an instrumental platform for the amalgamation of data, yielding a diversified and voluminous dataset that enhanced the breadth of the chemical space explored. Such expansiveness in data is imperative to circumvent the limitations associated with narrowly defined applicability domains, addressing a prevailing challenge where QSAR models could potentially be rendered ineffective due to constrained coverage of chemical space. In this context, the hackathon emerged as an effective mechanism for gathering data and solutions, contributing to the enhancement of the models’ robustness and applicability in predictive toxicology.

## Figures and Tables

**Table 2:** Comparison of the toxicity parameter metrics for the MML model for five-fold cross-validation within a regression problem.

Toxicity Endpoint	Regression Task	
	RMSE_MML_	RMSE_benchmark_
Mouse oral LD_50_	**0.43**	0.49
Rat oral LD_50_	**0.47**	0.68
Mouse intraperitoneal LD_50_	**0.45**	0.54
Rat intraperitoneal LD_50_	**0.58**	0.64
Mouse intravenous LD_50_	**0.46**	0.52
Rat intravenous LD_50_	**0.59**	0.63

**Table 3:** ROC AUC values for five-fold cross-validation for the Billy QSAR and SOTA language models [45].

Toxicity Endpoint	Billy QSAR	Smi2Vec-LSTM	Smi2Vec-BiGRU	TranGRU
NR.AhR ^1^	**0.904**	0.678	0.879	0.833
NR.AR ^1^	0.771	0.691	0.714	**0.824**
NR.AR.LBD ^1^	0.747	0.748	0.824	**0.847**
NR.Aromatase	**0.802**	0.496	0.699	0.784
NR.ER ^1^	**0.787**	0.623	0.736	0.691
NR.ER.LBD ^1^	0.763	0.531	**0.868**	0.843
NR.PPAR.gamma ^1^	0.767	0.566	0.749	**0.838**
SR.ARE ^1^	**0.795**	0.641	0.761	0.701
SR.ATAD5 ^1^	**0.806**	0.5	0.763	0.727
SR.HSE ^1^	**0.796**	0.612	0.785	0.736
SR.MMP ^1^	**0.951**	0.743	0.86	0.816
SR.p53	**0.818**	0.518	0.732	0.81

^1^ NR.AhR—aryl hydrocarbon receptor; NR.AR—androgen receptor; NR.AR.LBD—androgen receptor ligand-binding domain; NR.ER—estrogen receptor; NR.ER.LBD—estrogen receptor ligand-binding domain; NR.PPAR.gamma—peroxisome proliferator-activated receptor gamma; SR.ARE—antioxidant responsive element; SR.ATAD5—ATPase family AAA domain containing 5 gene; SR.HSE—stress response heat shock sequence; SR.MMP—stress response mitochondrial membrane potential.

**Table 4:** RMSE values for five-fold cross-validation for all targets from the hackathon with our models using gradient boosting and model metrics from the literature.

Target Name	CatBoost Fingerprints	XGBoost Fragments	Benchmark *	*n* Samples
Mouse intraperitoneal LD_50_	0.562	0.486	**0.473** (TOXRIC)	91,162
Mouse oral LD_50_	0.543	**0.445**	**0.445** (TOXRIC)	57,307
Mouse intravenous LD_50_	0.498	**0.415**	0.491 (TOXRIC)	41,630
Rat oral LD_50_	0.589	**0.466**	0.592 (Syntelly)	23,409
Mouse subcutaneous LD_50_	0.696	0.652	**0.55** (Syntelly)	19,457
Rat intraperitoneal LD_50_	0.71	0.618	**0.61** (Syntelly)	12,769
Rat intravenous LD_50_	0.894	0.853	**0.644** (TOXRIC)	7461
Rat subcutaneous LD_50_	0.829	0.743	**0.69** (Syntelly)	5376
Tetrahymena pyriformis IGC_50_ 40 h	0.524	**0.46**	0.518 (TOXRIC)	3516
Mouse intraperitoneal LD_Lo_	0.495	**0.435**	0.52 (Syntelly)	3500
Rabbit skin LD_50_	0.521	**0.479**	0.58 (Syntelly)	3429
Rabbit oral LD_50_	0.626	**0.588**	**0.588** (Syntelly)	2969
Guinea pig oral LD_50_	0.703	**0.659**	0.69 (Syntelly)	1778
Fathead minnow LC_50_ 96 h	0.78	**0.72**	0.864 (TOXRIC)	1739
Rat skin LD_50_	0.665	0.63	**0.62** (Syntelly)	1673
Rabbit intravenous LD_50_	0.898	0.83	**0.67** (Syntelly)	1604
Rat intraperitoneal LD_Lo_	0.512	**0.482**	0.63 (Syntelly)	1568
Mouse intramuscular LD_50_	0.894	0.866	**0.715** (TOXRIC)	1518
Rat oral LD_Lo_	0.997	0.99	**0.71** (Syntelly)	1464
Bioconcentration factor	0.699	**0.626**	0.71 (Syntelly)	1321
Dog intravenous LD_50_	0.995	0.927	**0.838** (TOXRIC)	1215
Chicken oral LD_50_	**0.906**	0.941	0.916 (TOXRIC)	743
Quail oral LD_50_	**0.816**	0.852	0.817 (TOXRIC)	735
Dog intravenous LD_Lo_	0.84	**0.834**	0.894 (TOXRIC)	703
Daphnia magna LC_50_	0.866	**0.817**	1.109 (TOXRIC)	699
Rabbit intravenous LD_Lo_	0.783	**0.749**	1.031 (TOXRIC)	690
Guinea pig intraperitoneal LD_50_	**0.745**	0.786	0.818 (TOXRIC)	631
Cat intravenous LD_50_	0.877	0.851	**0.836** (TOXRIC)	542
Mouse skin LD_50_	0.838	**0.815**	0.917 (TOXRIC)	426

* RMSE values were selected from the Syntelly [44] or TOXRIC [48] websites.

**Table 5:** ROC AUC values for five-fold cross-validation for all targets that were mentioned in the hackathon.

Target Name	CatBoost Fingerprints	XGBoost Fragments	Benchmark ^1^	*n* Samples
Cardiotoxicity (hERG binary)	0.888	0.926	**0.930** (CardioTox)	324,010
Ames test	0.845	**0.894**	0.88 (Syntelly)	14,168
SR-HSE ^2^	**0.839**	0.836	0.736 (TranGRU)	7281
NR-AR ^2^	0.724	0.797	**0.824** (TranGRU)	7263
NR-AR-LBD ^2^	0.843	0.835	**0.847** (TranGRU)	7133
NR-PPAR-gamma ^2^	0.727	0.809	**0.838** (TranGRU)	6942
NR-aromatase	0.831	**0.875**	0.784 (TranGRU)	6929
NR-ER-LBD ^2^	0.858	**0.878**	0.843 (TranGRU)	6920
SR-ATAD5 ^2^	0.75	**0.862**	0.727 (TranGRU)	6893
SR-ARE ^2^	0.825	**0.845**	0.701 (TranGRU)	6822
SR-p53	0.748	**0.877**	0.81 (TranGRU)	6749
NR-ER ^2^	0.852	**0.866**	0.691 (TranGRU)	6585
NR-AhR ^2^	0.816	**0.871**	0.833 (TranGRU)	6446
SR-MMP ^2^	0.853	**0.896**	0.816 (TranGRU)	6361
Eye irritation	0.977	**0.98**	0.966 (TOXRIC)	5040
Hepatotoxicity	0.785	**0.811**	0.741 (TOXRIC)	3413
Carcinogenicity	0.757	**0.787**	0.68 (TOXRIC)	2726
Eye corrosion	**0.993**	0.99	0.948 (TOXRIC)	2190
Blood–brain barrier penetration	0.925	**0.936**	0.919 (Wang et al.)	1961
Developmental toxicity	0.85	0.857	**0.918** (TOXRIC)	640
DILI ^2^	0.874	**0.901**	0.691 (Lim et al.)	475
Reproductive toxicity	0.489	0.739	**0.927** (TOXRIC)	146

^1^ ROC AUC values were selected from publications [45,49,50,51] or the Syntelly [44] or TOXRIC [48] websites. ^2^ SR-HSE—stress response heat shock sequence; NR-AR—androgen receptor; NR-AR-LBD—androgen receptor ligand-binding domain; NR-PPAR-gamma—peroxisome proliferator-activated receptor gamma; NR-ER-LBD—estrogen receptor ligand-binding domain; SR-ATAD5—ATPase family AAA domain containing 5 gene; SR-ARE—antioxidant responsive element; NR-ER—estrogen receptor; NR-AhR—aryl hydrocarbon receptor; SR-MMP—stress response mitochondrial membrane potential; DILI—drug-induced liver injury.

**Table 6:** List of all toxicity sources and databases used at the hackathon.

Name of the Database	References
TOXRIC	[48]
BioScience DBC	[54]
EFSA	[55]
National Toxicology Program	[56]
COSMOS	[57]
EPA	[58]
OCHEM	[59]
TensorFlow	[60]
Cactus NIH	[61]
FDA	[62]
PubChem	[63]
Tox21	[64]
Carcinogenic Potency Database	[65]
GitHub	[66,67]
Publications	[35,68,69,70,71,72,73,74,75,76,77,78,79,80,81,82,83]
USGS	[84]
ChEMBL	[85]
Kaggle	[86]
PyTDC	[87]
WeiLab MSU	[88]
CompTox	[89]
NLM NIH	[90,91]
SIDER	[92]
DrugBank	[93]
NORMAN	[94]
T3DB	[95]

## Data Availability

Data are contained within the article and Supplementary Materials.

## Supplementary Materials

The following supporting information can be downloaded at https://www.mdpi.com/article/10.3390/molecules29081826/s1. S1. Hyperparameter optimization. Table S1. Division into groups of features that were used by participants during the hackathon. Table S2. Abbreviations for the molecular descriptors. References [99,100,101,102] are cited in the Supplementary Materials.

## References

[1] Hornberg J.J., Laursen M., Brenden N., Persson M., Thougaard A.V., Toft D.B., Mow T. (2014). Exploratory toxicology as an integrated part of drug discovery. Part I: Why and how. Drug Discov. Today.

[2] Kong D.-X., Li X.-J., Zhang H.-Y. (2009). Where is the hope for drug discovery? Let history tell the future. Drug Discov. Today.

[3] Taglang G., Jackson D.B. (2016). Use of “big data” in drug discovery and clinical trials. Gynecol. Oncol..

[4] Kell D.B. (2013). Finding novel pharmaceuticals in the systems biology era using multiple effective drug targets, phenotypic screening and knowledge of transporters: Where drug discovery went wrong and how to fix it. FEBS J..

[5] Thomas C.E., Will Y. (2012). The impact of assay technology as applied to safety assessment in reducing compound attrition in drug discovery. Expert. Opin. Drug Discov..

[6] Aghila Rani K.G., Hamad M.A., DZaher M., Sieburth S.M., Madani N., Al-Tel T.H. (2021). Drug development post COVID-19 pandemic: Toward a better system to meet current and future global health challenges. Expert. Opin. Drug Discov..

[7] Amir-Aslani A. (2008). Toxicogenomic predictive modeling: Emerging opportunities for more efficient drug discovery and development. Technol. Forecast. Social. Change.

[8] Li A.P. (2009). Overview: Evaluation of metabolism-based drug toxicity in drug development. Chem. Biol. Interact..

[9] Ji C., Svensson F., Zoufir A., Bender A. (2018). eMolTox: Prediction of molecular toxicity with confidence. Bioinformatics.

[10] Roncaglioni A., Toropov A.A., Toropova A.P., Benfenati E. (2013). In silico methods to predict drug toxicity. Curr. Opin. Pharmacol..

[11] Wathieu H., Ojo A., Dakshanamurthy S. (2017). Prediction of Chemical Multi-target Profiles and Adverse Outcomes with Systems Toxicology. Curr. Med. Chem..

[12] Chen L., Lu J., Zhang J., Feng K.-R., Zheng M.-Y., Cai Y.-D. (2013). Predicting chemical toxicity effects based on chemical-chemical interactions. PLoS ONE.

[13] Jain S., Siramshetty V.B., Alves V.M., Muratov E.N., Kleinstreuer N., Tropsha A., Nicklaus M.C., Simeonov A., Zakharov A.V. (2021). Large-Scale Modeling of Multispecies Acute Toxicity End Points Using Consensus of Multitask Deep Learning Methods. J. Chem. Inf. Model..

[14] Sushko I., Novotarskyi S., Körner R., Pandey A.K., Rupp M., Teetz W., Brandmaier S., Abdelaziz A., Prokopenko V.V., Tanchuk V.Y. (2011). Online chemical modeling environment (OCHEM): Web platform for data storage, model development and publishing of chemical information. J. Comput. Aided Mol. Des..

[15] Wang M.W.H., Goodman J.M., Allen T.E.H. (2021). Machine Learning in Predictive Toxicology: Recent Applications and Future Directions for Classification Models. Chem. Res. Toxicol..

[16] Watanabe H., Tamura I., Abe R., Takanobu H., Nakamura A., Suzuki T., Hirose A., Nishimura T., Tatarazako N. (2016). Chronic toxicity of an environmentally relevant mixture of pharmaceuticals to three aquatic organisms (alga, daphnid, and fish). Environ. Toxicol. Chem..

[17] Bell S.M., Phillips J., Sedykh A., Tandon A., Sprankle C., Morefield S.Q., Shapiro A., Allen D., Shah R., Maull E.A. (2017). An Integrated Chemical Environment to Support 21st-Century Toxicology. Environ. Health Perspect..

[18] Tice R.R., Austin C.P., Kavlock R.J., Bucher J.R. (2013). Improving the Human Hazard Characterization of Chemicals: A Tox21 Update. Environ. Health Perspect..

[19] Judson R.S., Houck K.A., Kavlock R.J., Knudsen T.B., Martin M.T., Mortensen H.M., Reif D.M., Rotroff D.M., Shah I., Richard A.M. (2010). In vitro screening of environmental chemicals for targeted testing prioritization: The ToxCast project. Environ. Health Perspect..

[20] Ginsberg G.L., Fedinick K.P., Solomon G.M., Elliott K.C., Vandenberg J.J., Barone S., Bucher J.R. (2019). New Toxicology Tools and the Emerging Paradigm Shift in Environmental Health Decision-Making. Environ. Health Perspect..

[21] Kirchhübel N., Fantke P. (2019). Getting the chemicals right: Toward characterizing toxicity and ecotoxicity impacts of inorganic substances. J. Clean. Prod..

[22] Olker J.H., Elonen C.M., Pilli A., Anderson A., Kinziger B., Erickson S., Skopinski M., Pomplun A., LaLone C.A., Russom C.L. (2022). The ECOTOXicology Knowledgebase: A Curated Database of Ecologically Relevant Toxicity Tests to Support Environmental Research and Risk Assessment. Enviro Toxic. Chem..

[23] Wignall J.A., Muratov E., Sedykh A., Guyton K.Z., Tropsha A., Rusyn I., Chiu W.A. (2018). Conditional Toxicity Value (CTV) Predictor: An In Silico Approach for Generating Quantitative Risk Estimates for Chemicals. Environ. Health Perspect..

[24] LeBlanc G.A., Olmstead A.W. (2004). Evaluating the Toxicity of Chemical Mixtures. Environ. Health Perspect..

[25] Kramer C., Dahl G., Tyrchan C., Ulander J. (2016). A comprehensive company database analysis of biological assay variability. Drug Discov. Today.

[26] Price P.S., Keenan R.E., Swartout J.C. (2008). Characterizing interspecies uncertainty using data from studies of anti-neoplastic agents in animals and humans. Toxicol. Appl. Pharmacol..

[27] Szabó B., Lang Z., Kövér S., Bakonyi G. (2021). The inter-individual variance can provide additional information for the ecotoxicologists beside the mean. Ecotoxicol. Environ. Saf..

[28] Daina A., Michielin O., Zoete V. (2017). Zoete, SwissADME: A free web tool to evaluate pharmacokinetics, drug-likeness and medicinal chemistry friendliness of small molecules. Sci. Rep..

[29] Dopazo J. (2014). Genomics and transcriptomics in drug discovery. Drug Discovery Today.

[30] Hsieh J.-H., Yin S., Wang X.S., Liu S., Dokholyan N.V., Tropsha A. (2012). Cheminformatics meets molecular mechanics: A combined application of knowledge-based pose scoring and physical force field-based hit scoring functions improves the accuracy of structure-based virtual screening. J. Chem. Inf. Model..

[31] RDKit. https://www.rdkit.org/.

[32] D3R|D3R Grand Challenge. https://drugdesigndata.org/about/grand-challenge.

[33] Ferguson A.L., Mueller T., Rajasekaran S., Reich B.J. (2019). Conference report: 2018 materials and data science hackathon (MATDAT18). Mol. Syst. Des. Eng..

[34] Drugathon 2023 • BioSolveIT. https://www.biosolveit.de/drugathon-2023/.

[35] Mansouri K., Karmaus A.L., Fitzpatrick J., Patlewicz G., Pradeep P., Alberga D., Alepee N., Allen T.E., Allen D., Alves V.M. (2021). CATMoS: Collaborative Acute Toxicity Modeling Suite. Environ. Health Perspect..

[36] Kaggle. https://www.kaggle.com/.

[37] Nomad2018 Predicting Transparent Conductors. https://kaggle.com/competitions/nomad2018-predict-transparent-conductors.

[38] Novozymes Enzyme Stability Prediction. https://kaggle.com/competitions/novozymes-enzyme-stability-prediction.

[39] Predicting Molecular Properties. https://kaggle.com/competitions/champs-scalar-coupling.

[40] Bristol-Myers Squibb—Molecular Translation. https://kaggle.com/competitions/bms-molecular-translation.

[41] Syntelly Hackathon. https://syntelly.ru/russianmedia/tpost/g0ainxvja1-obyavleni-pobediteli-hakatona.

[42] CatBoost. https://catboost.ai/.

[43] Sosnin S., Karlov D., Tetko I.V., Fedorov M.V. (2019). Comparative Study of Multitask Toxicity Modeling on a Broad Chemical Space. J. Chem. Inf. Model..

[44] Syntelly. https://app.syntelly.com/login.

[45] Jiang J., Zhang R., Ma J., Liu Y., Yang E., Du S., Zhao Z., Yuan Y. (2023). TranGRU: Focusing on both the local and global information of molecules for molecular property prediction. Appl. Intell..

[46] Choudhary K., DeCost B. (2021). Atomistic Line Graph Neural Network for improved materials property predictions. npj Comput. Mater..

[47] XGBoost. https://xgboost.readthedocs.io/en/stable/#.

[48] Wu L., Yan B., Han J., Li R., Xiao J., He S., Bo X. (2023). TOXRIC: A comprehensive database of toxicological data and benchmarks. Nucleic Acids Res..

[49] Karim A., Lee M., Balle T., Sattar A. (2021). CardioTox net: A robust predictor for hERG channel blockade based on deep learning meta-feature ensembles. J. Cheminform..

[50] Wang Z., Yang H., Wu Z., Wang T., Li W., Tang Y., Liu G. (2018). In Silico Prediction of Blood-Brain Barrier Permeability of Compounds by Machine Learning and Resampling Methods. ChemMedChem.

[51] Lim S., Kim Y., Gu J., Lee S., Shin W., Kim S. (2023). Supervised chemical graph mining improves drug-induced liver injury prediction. iScience.

[52] LightGBM. https://lightgbm.readthedocs.io/en/stable/.

[53] Boldini D., Grisoni F., Kuhn D., Friedrich L., Sieber S.A. (2023). Practical guidelines for the use of gradient boosting for molecular property prediction. J. Cheminformatics.

[54] BioScience Database. https://dbarchive.biosciencedbc.jp/index.html.

[55] EFSA (European Food Safety Authority) Chemical Hazards Database—OpenFoodTox. https://www.efsa.europa.eu/en/data-report/chemical-hazards-database-openfoodtox.

[56] CEBS (Chemical Effects in Biological Systems). https://cebs.niehs.nih.gov/cebs/.

[57] Cosmos D.B. https://www.ng.cosmosdb.eu/downloads.

[58] EPA Ecotox Database. https://cfpub.epa.gov/ecotox/.

[59] OCHEM Database. https://ochem.eu/home/show.do.

[60] TensorFlow Datasets. https://www.tensorflow.org/datasets.

[61] NCI CACTUS Chemical Identifier Search. https://cactus.nci.nih.gov/index.html.

[62] FDA Drug-Induced Liver Injury Rank (DILIrank) Dataset. https://fda.gov/science-research/liver-toxicity-knowledge-base-ltkb/drug-induced-liver-injury-rank-dilirank-dataset.

[63] PubChem Database. https://pubchem.ncbi.nlm.nih.gov/.

[64] Richard A.M., Huang R., Waidyanatha S., Shinn P., Collins B.J., Thillainadarajah I., Grulke C.M., Williams A.J., Lougee R.R., Judson R.S. (2021). The Tox21 10K Compound Library: Collaborative Chemistry Advancing Toxicology. Chem. Res. Toxicol..

[65] NLM CPDB. https://www.nlm.nih.gov/index.html.

[66] Molecules Dataset Collection. https://github.com/GLambard/Molecules_Dataset_Collection.

[67] CardioTox. https://github.com/Abdulk084/CardioTox.

[68] Wu K., Wei G.-W. (2018). Quantitative Toxicity Prediction Using Topology Based Multitask Deep Neural Networks. J. Chem. Inf. Model..

[69] Lagunin A., Filimonov D., Zakharov A., Xie W., Huang Y., Zhu F., Shen T., Yao J., Poroikov V. (2009). Computer-Aided Prediction of Rodent Carcinogenicity by PASS and CISOC-PSCT. QSAR Comb. Sci..

[70] Lee H.-M., Yu M.-S., Kazmi S.R., Oh S.Y., Rhee K.H., Bae M.A., Lee B.H., Shin D.S., Oh K.S., Ceong H. (2019). Computational Determination of hERG-Related Cardiotoxicity of Drug Candidates. BMC Bioinform..

[71] Shen M.-Y., Su B.-H., Esposito E.X., Hopfinger A.J., Tseng Y.J. (2011). A Comprehensive Support Vector Machine Binary hERG Classification Model Based on Extensive but Biased End Point hERG Data Sets. Chem. Res. Toxicol..

[72] Wang S., Sun H., Liu H., Li D., Li Y., Hou T. (2016). ADMET Evaluation in Drug Discovery. 16. Predicting hERG Blockers by Combining Multiple Pharmacophores and Machine Learning Approaches. Mol. Pharm..

[73] Xu Y., Dai Z., Chen F., Gao S., Pei J., Lai L. (2015). Deep Learning for Drug-Induced Liver Injury. J. Chem. Inf. Model..

[74] Cai C., Guo P., Zhou Y., Zhou J., Wang Q., Zhang F., Fang J., Cheng F. (2019). Deep Learning-Based Prediction of Drug-Induced Cardiotoxicity. J. Chem. Inf. Model..

[75] Hansen K., Mika S., Schroeter T., Sutter A., ter Laak A., Steger-Hartmann T., Heinrich N., Müller K.-R. (2009). Benchmark Data Set for in Silico Prediction of Ames Mutagenicity. J. Chem. Inf. Model..

[76] Wu Z., Jiang D., Wang J., Hsieh C.-Y., Cao D., Hou T. (2021). Mining Toxicity Information from Large Amounts of Toxicity Data. J. Med. Chem..

[77] Braga R.C., Alves V.M., Silva M.F., Muratov E., Fourches D., Lião L.M., Tropsha A., Andrade C.H. (2015). Pred-hERG: A Novel Web-Accessible Computational Tool for Predicting Cardiac Toxicity. Mol. Inform..

[78] Kumar R., Sharma A., Alexiou A., Bilgrami A.L., Kamal M.A., Ashraf G.M. (2022). DeePred-BBB: A Blood Brain Barrier Permeability Prediction Model with Improved Accuracy. Front. Neurosci..

[79] Martins I.F., Teixeira A.L., Pinheiro L., Falcao A.O. (2012). A Bayesian Approach to in Silico Blood-Brain Barrier Penetration Modeling. J. Chem. Inf. Model..

[80] Tong X., Wang D., Ding X., Tan X., Ren Q., Chen G., Rong Y., Xu T., Huang J., Jiang H. (2022). Blood–brain Barrier Penetration Prediction Enhanced by Uncertainty Estimation. J. Cheminform..

[81] Feinstein J., Sivaraman G., Picel K., Peters B., Vázquez-Mayagoitia, Ramanathan A., MacDonell M., Foster I., Yan E. (2021). Uncertainty-Informed Deep Transfer Learning of Perfluoroalkyl and Polyfluoroalkyl Substance Toxicity. J. Chem. Inf. Model..

[82] Li P., Li Y., Hsieh C.-Y., Zhang S., Liu X., Liu H., Song S., Yao X. (2021). TrimNet: Learning Molecular Representation from Triplet Messages for Biomedicine. Brief. Bioinform..

[83] Meng F., Xi Y., Huang J., Ayers P.W. (2021). A Curated Diverse Molecular Database of Blood-Brain Barrier Permeability with Chemical Descriptors. Sci. Data..

[84] Acute Toxicity Data. https://www.cerc.usgs.gov/data/acute/acute.html.

[85] ChEMBL Database. https://www.ebi.ac.uk/chembl/.

[86] BBBP-SMILES Dataset on Kaggle. https://www.kaggle.com/datasets/priyanagda/bbbp-smiles.

[87] WeiLab Mathematical Data Library. https://weilab.math.msu.edu/DataLibrary/2D/.

[88] CompTox Chemicals Dashboard. https://www.epa.gov/chemical-research/comptox-chemicals-dashboard.

[89] LactMed Database. https://www.nlm.nih.gov/databases/download/lactmed.html.

[90] CCRIS Database. https://www.nlm.nih.gov/databases/download/ccris.html.

[91] DrugBank Online. https://go.drugbank.com/.

[92] NORMAN Network Data System. https://www.norman-network.com/nds/SLE/.

[93] Huang K., Fu T., Gao W., Zhao Y., Roohani Y., Leskovec J., Coley C.W., Xiao C., Sun J., Zitnik M. (2022). Artificial Intelligence Foundation for Therapeutic Science. Nat. Chem. Biol..

[94] Kuhn M., Letunic I., Jensen L.J., Bork P. (2016). The SIDER Database of Drugs and Side Effects. Nucleic Acids Res..

[95] Wishart D., Arndt D., Pon A., Sajed T., Guo A.C., Djoumbou Y., Knox C., Wilson M., Liang Y., Grant J. (2015). T3DB: The Toxic Exposome Database. Nucleic Acids Res..

[96] Alves V.M., Muratov E.N., Capuzzi S.J., Politi R., Low Y., Braga R.C., Zakharov A.V., Sedykh A., Mokshyna E., Farag S. (2016). Alarms about structural alerts. Green. Chem..

[97] SLiang S.-T., Chen C., Chen R.-X., Li R., Chen W.-L., Jiang G.-H., Du L.-L. (2022). Michael acceptor molecules in natural products and their mechanism of action. Front. Pharmacol..

[98] Limban C., Nuţă D.C., Chiriţă C., Negreș S., Arsene A.L., Goumenou M., Karakitsios S.P., Tsatsakis A.M., Sarigiannis D.A. (2018). The use of structural alerts to avoid the toxicity of pharmaceuticals. Toxicol. Rep..

[99] Prasanna S., Doerksen R.-J. (2009). Topological polar surface area: A useful descriptor in 2D-QSAR. Curr. Med. Chem..

[100] Labute P. (2000). A widely applicable set of descriptors. J. Mol. Graph Model.

[101] Kier L., Hall L. (1991). A Differential Molecular Connectivity Index. Quant. Struct. Act. Relatsh..

[102] Menchinskaya E., Chingizova E., Pislyagin E., Likhatskaya G., Sabutski Y., Pelageev D. (2021). europrotective Effect of 1,4-Naphthoquinones in an In Vitro Model of Paraquat and 6-OHDA-Induced Neurotoxicity. Int. J. Mol. Sci..

