# Supplementary material for: Expanding Predictive Capacities in Toxicology: Insights from Hackathon-Enhanced Data and Model Aggregation
Source: Molecules. 2024 Apr 17;29(8):1826. doi: 10.3390/molecules29081826 (PMC11055041; doi:10.3390/molecules29081826)
Supplement: Supplementary file 1 [file molecules-29-01826-s001.zip › molecules-2885307-supplementary.pdf]

Supporting Information

Expanding Predictive Capacities in Toxicology: Insights from Hackathon-Enhanced Data and Model Aggregation

Dmitrii O. Shkil [1,2,*], Alina A. Muhamedzhanova [1], Philipp I. Petrov [3], Ekaterina V. Skorb [4], Timur A. Aliev [4],
Ilya S. Steshin [1], Alexander V. Tumanov [1], Alexander S. Kislinskiy [1] and Maxim V. Fedorov [5,*]

[1] Syntelly LLC, Moscow 121205, Russia; muhamedzhanova@syntelly.com (A.A.M.); steshin@syntelly.com (I.S.S.); tumanov@syntelly.com (A.V.T.); kislinskiy@syntelly.com (A.S.K.)
[2] Moscow Institute of Physics and Technology, Moscow 141700, Russia
[3] Medtech.Moscow, Moscow 119571, Russia; philip.i.petrov@gmail.com
[4] Infochemistry Scientific Center, ITMO University, Saint-Petersburg 191002, Russia; skorb@itmo.ru (E.V.S.); aliev@infochemistry.ru (T.A.A.)
[5] Kharkevich Institute for Information Transmission Problems of Russian Academy of Sciences,
Moscow 127994, Russia
* Correspondence: shkil@syntelly.com (D.O.S.); fedorov@iitp.ru (M.V.F.)

S1. Hyperparameter optimization.

For CatBoost, Optuna hyperparameter search was used for each of the hackathon datasets:

1. learning_rate: range(10^{-5}, 1, log=True);
2. iterations: range(100, 1000);
3. colsample_bylevel: range(0.01, 0.1);
4. l2_leaf_reg: range(10^{-8}, 100, log=True);
5. depth: range(3, 11);
6. random_strength: range(10^{-7}, 20.0, log=True);
7. boosting_type: [Ordered, Plain];
8. bootstrap_type: [Bayesian, Bernoulli, MVS];
9. bagging_temperature: range(0, 10);
10. subsample: range(0.1, 1).

For XGBoost, a grid search was carried out for regression problems (Mouse Intraperitoneal LD_{50}, Mouse Intravenous LD_{50}, Mouse Oral LD_{50}) and classification (BBB Penetration). Among the gridsearch parameters we used:

1. max_depth: range(1, 14, 1);
2. learning_rate: [0.1, 0.01, 0.001];
3. subsample: [1, 0.75, 0.5, 0.3];
4. n_estimators: [1500, 2000, 2500, 3000];
5. reg_lambda: [1, 2, 4];
6. reg_alpha: [0, 10, 40];
7. colsample_bytree: [1, 0.75, 0.5, 0.3];
8. min_child_weight: [1, 5, 10].

The best parameters from gridsearch were selected: learning_rate=0.01, n_estimators=2000, max_depth=12. Tree construction method: approximate greedy algorithm optimized for histogram (hist). For the remaining datasets, a reduced gridsearch subsample was used: [1, 0.75, 0.5, 0.3]. Reduced gridsearch was used to save hyperparameter selection time. Another reason that there was no actual difference between selected hyperparameters and founded paramaters for particular toxicity case, except subsample value.

Descriptors

Table S1. Divided into groups features that were used by participants during the hackathon.

Descriptors	Fingerprints	Graph Featurizers	Text Embeddings
CATS	Avalon	DeepChem	ChemBERT
Mordred	Ghose Crippen	PyTorch Geometric	PyTorch
Murcko Scaffold	MACCS	graph2vec	RoBERTa
PaDELPy	Morgan	node2vec	Sklearn TF-IDF
PyBioMed	PaDELPy		mol2vec
RDKit			

Table S2. Used abbreviations for molecular descriptors

Descriptor	Decipherment	Link
TPSA	Topological polar surface area based on fragments	[1]
LabuteASA	Labute's Approximate Surface Area	[2]
Kappa 1-3	A differential molecular connectivity index	[3]
SlogPVSA	MOE-type descriptor using SLogP contributions and surface area contributions	[4]
SMRVSA	MOE-type descriptor using molar refractivity contributions and surface area contributions	[4]
EStateVSA	Electron State Van der Waals Surface Area Descriptor	[2]

References

1. Prasanna, S.; Doerksen, R.-J. Topological polar surface area: a useful descriptor in 2D-QSAR. *Curr. Med. Chem.* **2009**, *16*, 21–41.
2. Labute, P. A widely applicable set of descriptors. *J. Mol. Graph. Model* **2000**, *18*, 464–477.
3. Kier, L.; Hall, L. A Differential Molecular Connectivity Index. *Quant. Struct. Act. Relatsh.* **1991**, *10*, 134–140.
4. Menchinskaya, E.; Chingizova, E.; Pislyagin, E.; Likhatskaya, G.; Sabutski, Y.; Pelageev, D.; Plolonik, S.; Aminin, D. Neuroprotective Effect of 1,4-Naphthoquinones in an In Vitro Model of Paraquat and 6-OHDA-Induced Neurotoxicity. *Int. J. Mol. Sci.* **2021**, *22*, 9933.
